# The relationship between self-esteem and bullying behavior among adolescent in tunisia

**DOI:** 10.1192/j.eurpsy.2021.576

**Published:** 2021-08-13

**Authors:** R. Ayoub, T. Brahim, N. Ben Salem, N. Brigui, A. Guedria, N. Gaddour

**Affiliations:** 1 Child And Adolescent Psychiatry Department, Fattouma Bourguiba University Hospital, Monastir, Tunisia; 2 Pedopsychiatrie, fattouma bourguiba hospital, monastir, Tunisia

**Keywords:** self-esteem, bullying behavior, adolescent

## Abstract

**Introduction:**

Bullying is a serious problem for school youth. It is prevalent across the elementary and secondary school years and it has serious consequences for both bullies and victims.

**Objectives:**

The aim of this study was to investigate the relationship between self-esteem and bullying behavior.

**Methods:**

We conducted a cross-sectional study including children enrolled in two high schools in Sousse, Tunisia. The students were asked to complete two questionnaires: the Adolescent Peer Relations Instrument witch is a multidimensional scale designed to assess bullying involvement both as target and perpetrator and the Rosenberg self-esteem scale.

**Results:**

We recruited 600 adolescent. The mean age of our population was 13.76 ±1.37 and the sex ratio was 1. More than 95% of adolescent who reported that they had been victims of bullying had a very low self-esteem comparing to those who stated that they had never been bullied (4.4%). Our results have also shown that bullies had a lower self-esteem than children who had not bullied others.

**Conclusions:**

We found that both victims and bullies tend to have low self-esteem. Our findings could help to understand better the role that individual characteristics and personal qualities such as self-esteem play on bullying, and provide the scientific knowledge to develop successful strategies to prevent this phenomenon.

